# Evaluate construct validity of the Revised American Pain Society Patient Outcome Questionnaire in gynecological postoperative patients using confirmatory factor analysis

**DOI:** 10.1186/s12871-020-01229-x

**Published:** 2021-01-15

**Authors:** Sook Hui Chaw, Yoke Lin Lo, Jia Yin Lee, Jia Wing Wong, Wan Aizat Wan Zakaria, Shairil Rahayu Ruslan, Wei Keang Tan, Ina Ismiarti Shariffuddin

**Affiliations:** 1 Department of Anesthesiology, Faculty of Medicine, University of Malaya, Kuala Lumpur, Malaysia; 2 Department of Pharmacy Practice, School of Pharmacy, International Medical University, No 126 Jalan Jalil Perkasa 19, Bukit Jalil, Kuala Lumpur, Malaysia

**Keywords:** Construct validity, Factor analysis, Pain management, Patient management, Postoperative pain

## Abstract

**Background:**

The Revised American Pain Society Patient Outcome Questionnaire (APS-POQ-R) evaluates the patient-reported quality of pain management in adults. A validated APS-POQ-R is pivotal to guide effective pain management with better patient satisfaction. Previous studies revealed that subscales of “patients’ perception of pain management” were unstable cross-culturally. This study aims to evaluate the construct validity of the APS-POQ-R in gynecological postoperative patients with a multi-cultural background using confirmatory factor analysis to allow comparisons among different a priori models at the latent factor level.

**Methods:**

Patients aged 18 years old or above and who were scheduled for gynecology surgery were selected. Three different models with a combination of latent factors were based on a priori hypotheses from previous studies. The root-mean-squared error of approximation, comparative fit index, Tucker-Lewis Index, Chi-squared test, and change in Chi-squared statistic given a change in degrees of freedom between models were used to assess the model fit to the present data.

**Results:**

A total of 302 patients completed the questionnaire. The five-factor model which was based on Gordon’s study has an acceptable fit for the data and was superior when compared to the one-factor baseline model. Although the four-factor model, which originated from Botti’s study, also demonstrates a good model fit, the “perception of care” construct was excluded in this model. The “perception of care” construct is conceptually important as patient-centered care has become the focus of quality improvement of pain service.

**Conclusions:**

The APS-POQ-R is easy to administer and is useful for quality evaluation in postoperative pain management. The present study demonstrates that a five-factor structure of the APS-POQ-R is the best fitting model in our patient sample. The results of this study provide further evidence to support the use of APS-POQ-R as a measurement tool for pain management evaluation in acute postoperative patients with a multi-cultural background.

## Background

Pain is an inevitable consequence of surgery, and managing pain is a challenging task as pain is a subjective experience and multi-dimensional [1]. Postoperative pain is inadequately managed in more than 80% of surgical patients, and is associated with increased morbidity, impaired recovery from surgery, and decreased quality of life [2]. Besides monitoring of clinical outcomes, continual evaluation of the patient-reported outcome in the quality of pre- and post-operative pain management is pivotal to guide an effective health care delivery with a high level of patient satisfaction. For this purpose, a validated and standard quality improvement (QI) measure is essential.

The Acute Pain Society Patient Outcome Questionnaire (APS-POQ) was first developed in 1991 [3], and was subsequently revised in the years 1995 [4] and 2010 by the American Pain Society [5]. The Revised Acute Pain Society Patient Outcome Questionnaire (APS-POQ-R) could be a useful tool to measure the quality of pain management for QI purposes. The APS-POQ-R was originally organized into five factors in the psychometric evaluation by Gordon et al.: pain severity and sleep interference (5 items), activity interference (2 items), affection (4 items), adverse effects (4 items), and perceptions of care (3 items). While the original factor solution in that study showed a high degree of internal consistency, subsequent studies in various cohorts reviewed a range of factor solutions and patterns of item distribution [6–8].

Botti et al. divided the primary questionnaire items that were amenable to psychometric testing into two main categories based on their face meaning; namely, pain experience and patients’ perception of pain management. The “pain experience” category measures the pain severity, interference of physical and psychological well-being due to pain, and the “patients’ perception of pain management” category evaluates patients’ participation in decision making and their satisfaction [6]. The authors confirmed the validity and stability of APS-POQ-R to measure “pain experience” across different cultures. Nevertheless, the robustness of APS-POQ-R to measure “patients’ perception of pain management” across cultural groups was questioned due to the inconsistent loading of “pain management” items between different groups in their study. It is believed that the reasons for the variability in the perception of treatment across national groups are attributed to the difference in culture, health care delivery, language, and translation [6].

This finding was echoed in a study by Zoega et al. who evaluated the Icelandic version of the APS-POQ-R [8]. Although the initial principal components extracted in that study were consistent with those from Gordon et al. [5], the internal consistency obtained was unacceptable with a Cronbach α value of 0.42. To increase the internal consistency, Zoega et al. removed the perception of the care component in the final Icelandic version of APS-POQ-R [8]. The cross-cultural instability of APS-POQ-R to measure “patients’ perception of pain management” suggests the importance of conducting a validation evaluation of this questionnaire before implementing its use in a different regional setting.

From the literature, the models that were proposed for APS-OQ-R may have three-, four-, or five-factor structures. The differing patterns of item distribution and removal of items in previous studies render the questionnaire difficult to compare findings between cohorts [8, 9]. In this study, we aim to evaluate the construct validity of this instrument using the confirmatory factor analysis (CFA) framework of the previously proposed models in a multi-ethnic Malaysian population, focusing on patients receiving gynecological procedures. Confirmatory factor analysis, unlike exploratory factor analysis (EFA), allows comparisons of alternative a priori models proposed at the latent factor level to determine the best fitting model that describes the data [10]. Gynecological postoperative patients represent a large proportion of surgical patients and there is increasing evidence on sex differences in pain experience, and analgesia with women report greater pain in acute clinical pain settings [11]. With a validated QI instrument, we can explore the multi-dimension of pain experience in this group of patients and develop targeted pain management [12].

## Methods

This cross-sectional study was carried out at the University of Malaya Medical Centre (UMMC), a tertiary university hospital in Kuala Lumpur, Malaysia. The present study complies with the Declaration of Helsinki and the study protocol was approved by the UMMC Medical Research and Ethics Committee (Approval number: 2,016,122­4660), as well as the International Medical University Joint Committee on Research and Ethics (Approval number: IG544), Kuala Lumpur, Malaysia. Written informed consent from patients was obtained before their participation.

In our center, postoperative pain management is managed by both the Acute Pain Service (APS) team, and the primary surgical team. The usual postoperative multimodal analgesia regimens are a combination of opioids and non-opioid analgesics. The commonly used opioids include fentanyl, morphine, and oxycodone; while non-opioid analgesics are paracetamol, non-steroidal anti-inflammatory drugs (NSAIDs), and local anesthetic agents. Regional anesthetic techniques will be considered if indicated.

### Participants

Patients aged 18 years old or above and who were scheduled for elective gynecological surgery were eligible to be enrolled in this study. All elective surgeries were scheduled via the hospital’s online operation booking system in our center. A total of 320 patients were identified from the booking system. The investigator who was not directly involved in the management of the patients would communicate with them and introduce the APS-POQ-R questionnaire after they agreed to participate. A patient’s participation was voluntary, and standard care was ensured for all patients. We excluded the patients who had unexpected postoperative complications or intensive care admission as they were unable to answer the questionnaire.

### Survey instruments

The APS-POQ-R measures the quality of postoperative pain management among adults in the first 24 h of in-hospital patient care, and the questionnaire can be administered by interview or self-completion [5]. This questionnaire has been translated into 11 different languages and is widely used for QI purposes in pain management [3]. The APS-POQ-R can be obtained from the American Pain Society freely and can be used without further permission.

The questionnaire contains a total of 23 items which include 18 primary items (P1 – P9) and 3 secondary or information items (P10 – P12). The first three primary items (P1 – P3) measure patients’ pain intensity. Patients are asked to rate the least and the worst pain that they have in the first 24 h postoperatively on a numerical rating scale (NRS) ranging from 0 (No pain at all) to 10 (the worst pain possible). Meanwhile, item P3 examines the amount of time that patients experienced pain on a 0 to 100% scale during their first postoperative day.

Item P4 of the APS-POQ-R examines the interference of postoperative pain on patients’ routine functioning which includes activities in and out of bed, as well as the impact of pain on their sleep. Also, item P5 assesses the effects of pain that may result in anxiety, depression, fright, or helplessness on postoperative patients. Treatment-related adverse effects are an important component of pain management. Item P6 elicits the severity of side effects associated with analgesics. Insights regarding the side effects associated with postoperative pain management will help identify areas of improvement in the current practice.

Patients’ participation in the management of their care is of paramount importance to ensure comfort and to reduce potential complications [3]. The patients’ perception of the extent to which they can participate in decision-making in pain management is assessed in item P7. Item P8 grades the patients’ satisfaction in pain treatment received during the postoperative period. All items from P1 to P8 are measured with a numeric rating scale from 0 to 10, except for Item P3 and Item P7 which are measured in percentage.

The additional assessments on non-pharmacologic pain management include the items that evaluate whether the patients receive any information about other options for pain treatment, and how useful the information is to them (Item P9). The use of non-pharmacologic methods to relieve pain, and whether a nurse or a doctor encourages the patients to use non-pharmacologic methods are assessed in Item P10 and Item 11, respectively. These items give secondary information and are not included in the psychometric testing. The information on the patient characteristic profile is also obtained from the questionnaire.

### Data collection

The study was carried out between June 2017 and June 2018 using APS-POQ-R (English version). An investigator would approach potential study subjects preoperatively to obtain their informed consent. On a postoperative day, the consented patients were then asked to recall the pain management that they received in the first 24 h postoperatively. The demographic and clinical data of these patients were retrieved from their respective electronic medical records.

### Statistical analyses

#### Confirmatory factor analysis

We used confirmatory factor analysis (CFA) to evaluate the construct validity of the APS-POQ-R. The CFA was computed using maximum likelihood estimation in AMOS version 26™ (SPSS, IBM, Inc.). Confirmatory factor analysis is a subset of structural equation modeling (SEM), whereby it focuses on analyzing the extent to which the observed variables are related to the latent factors (measurement model); while SEM analyzes the causal relationship between observed variables and latent factors (structural model) [10]. Previous studies on APS-POQ-R utilized an exploratory factor analysis approach and showed various factor solutions in their respective settings. Confirmatory factor analysis reduces the numbers of observed variables to latent factors based on communalities and allows for comparisons of different a priori models at the latent factors level [13]. Using the CFA framework is suitable to fulfill the purpose of the present study: to find and confirm which constructs fit the data from our patient cohorts.

Multi-trait multimethod matrix (MTMM) is another statistical tool to evaluate construct validity. This analysis is used when the data set involves multiple constructs that are measured by different methods, such as self-report questionnaires, investigators’ observation, and so on [14]. Our study, however, used patient-report questionnaires to measure the underlying constructs. Thus, MTMM is not utilized in this study.

The models tested were based on the a priori hypothesis from previous studies [5, 6]. Four competing models were tested in the analysis. Model I, a single-factor model in which all 18 items were loaded on the latent variable, was used as a baseline comparison against the other models.

Model II, the original classification by Gordon et al. [5], was a five-factor model with items P1-P3 and P4c-P4d loaded on “Pain severity and sleep interference subscale”; items P5a-P5d loaded on “Affective subscale”; items P4a-P4b loaded on “Activity interference subscale”; items P6a-P6d loaded on “Adverse effect subscale”; and lastly, items P7-P9 loaded on “Perception of care subscale”. Model III was composed of four factors with items P1-P3, P4a-P4b, P7, and P9 loaded on “Pain severity/pain care and activity interference subscale”, P4c-P4d loaded on “Sleep interference subscale”, P5a-P5d loaded on “Affective subscale”, and P6a-P6d loaded on “Adverse effects subscale”. This model excludes item P8 (Ability to participate in pain treatment) that was based on the finding from Botti et al. [6], in which P8 did not load on any factors in the Danish cohort. Model IV, which was also a model based on Botti’s study [6], is a three-factor model with items P1-P3, P4a-P4d, and P7 loaded on “Pain severity/pain care and activity interference extended subscale”; P5 (a-d) and P6 (a-d) loaded on “Affect and adverse effects subscale”; and items in P8-P9 loaded on “Perception of care subscale”.

The assessment of model fit was based on several fit indices to test the best CFA model that describes the present data set and theoretical considerations. These indices include the root-mean-squared error of approximation (RMSEA), comparative fit index (CFI), Tucker-Lewis Index (TLI), Chi-squared test, and a change in Chi-squared statistic given a change in degrees of freedom between models. The RMSEA values closer to 0 with a threshold of less than 0.08 represent a good model fit [15]. The CFI and TLI values of 0.95 and above were considered indicative of an excellent fit; and above 0.9, is acceptable [16].

There is no easy number that one can use as a “large enough” sample size for structural equation modeling. Some rules of thumb are used; based on Kline et al., 100–200 observations are considered a medium sample size [17]. Different ratio rules are recommended, but for most multivariate analyses, the sample size should be at least 10 times the number of variables [18, 19]. With 18 items in the questionnaire that are subjected to factor analysis, a sample size of 320 is considered sufficient for psychometric testing in the present study.

#### Sample descriptive statistics

Descriptive statistics were used to describe the demographic data, and to report the results of the questionnaire items. Descriptive statistics were performed by using the Statistical Package for the Social Sciences, version 21.0 (SPSS Inc, Chicago, USA), and MS Excel (2013).

## Results

**Fig. 1:**
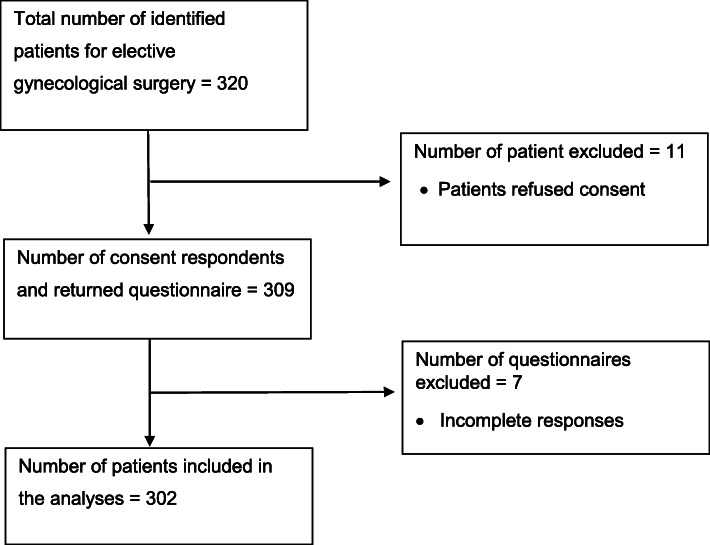
Flowchart of patient identification and recruitment processes according to STROBE guidelines

Of the 320 eligible patients in this study, 11 patients refused to participate, and seven returned questionnaires were incomplete. This gave a response rate of 94.4% (302/320). A flowchart of the patient recruitment process based on STROBE guidelines [20] is illustrated in Fig. 1. The demographics of the participants are depicted in Table 1.

**Table 1:** Baseline demographic data of the respondents (*N* = 302)

**Characteristics**	**Value**
**Age in years**, mean ± SD	48.7 ± 16.0
**Race**, n (%)	
Malay	141 (46.7%)
Chinese	87 (28.8%)
Indian	56 (18.5%)
Others	18 (5.9%)
**Religion**, n (%)	
Islam	159 (52.6%)
Buddhism	65 (21.5%)
Hinduism	47 (15.6%)
Christianity	31 (10.3%)
**Education level**, n (%)	
University	134 (44.4%)
Secondary school	118 (39.1%)
Elementary school	37 (12.3%)
No formal education	8 (2.6%)

*Abbreviations*: *N* Population size, *n* Sample size, *SD* Standard deviation

### Confirmatory factor analysis

**Fig. 2:**
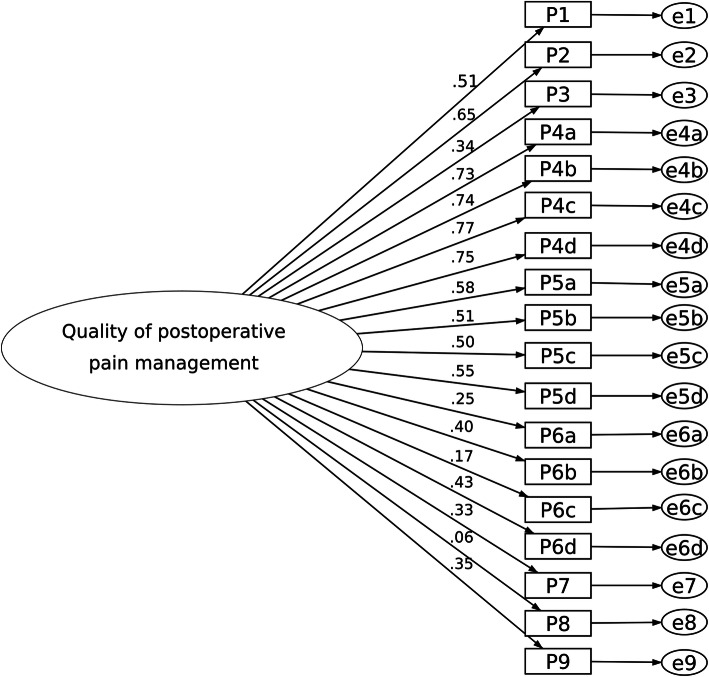
One-factor model (Model I) as a base model

**Fig. 3:**
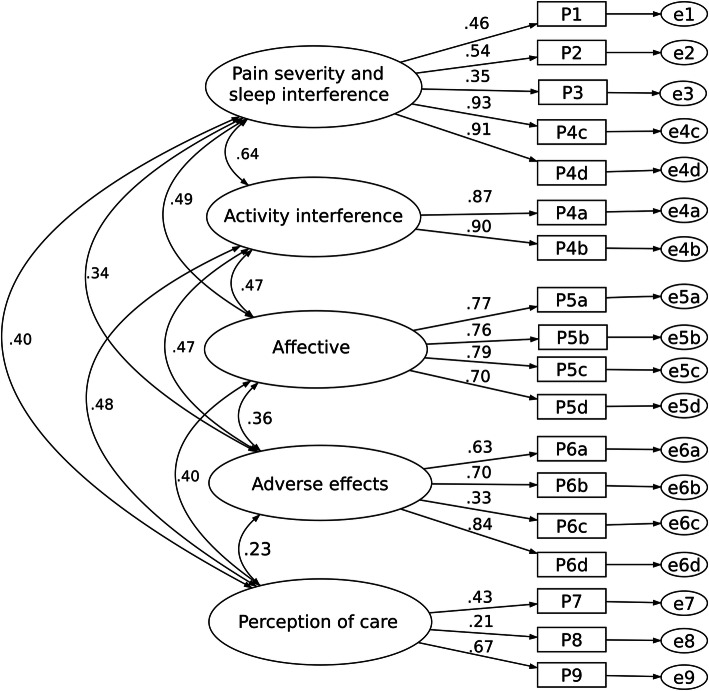
Five-factor model (Model II)

The fit statistics of all models are shown in Table 2. Figure 2 depicts the one-factor model as the base model. The model fit of the five-factor structure (Model II) as described in Gordon’s study fulfills an acceptable fit criterion (Fig. 3). The Chi-square score relative to degrees of freedom (χ^2^/df) is small (2.47), while the Comparative Fit Index (CFI) and Tucker-Lewis Index (TLI) are within an acceptable range. The root-mean-squared error of approximation (RMSEA) of this model also indicates an adequate fit.

**Table 2:** Fit indices for maximum likelihood confirmatory factor analyses between models

**Model**	**Model description**		**RMSEA (90% CI)**	**CFI**	**TFI**	**df**	**χ**^**2**^	**χ**^**2**^**/df**	***P***
Model I	One-factor model		0.146 (0.138, 0.155)	0.575	0.462	135	1005.34	7.45	
Model II	Five-factor model		0.070 (0.060, 0.080)	0.911	0.878	125	308.31	2.47	< 0.05
Model III	Four-factor model		0.068 (0.058, 0.079)	0.922	0.895	113	271.60	2.40	< 0.05
Model IV	Three-factor model		0.122 (0.113, 0.131)	0.711	0.626	132	723.66	5.482	< 0.05

*Abbreviations*: *RMSEA* Root-mean-square error of approximation, *CI* Confidence interval, *CFI* Comparative fit index, *TFI* Tucker-Lewis Index, *df* Degrees of freedom, *χ*^*2*^ Chi-square

**Fig. 4:**
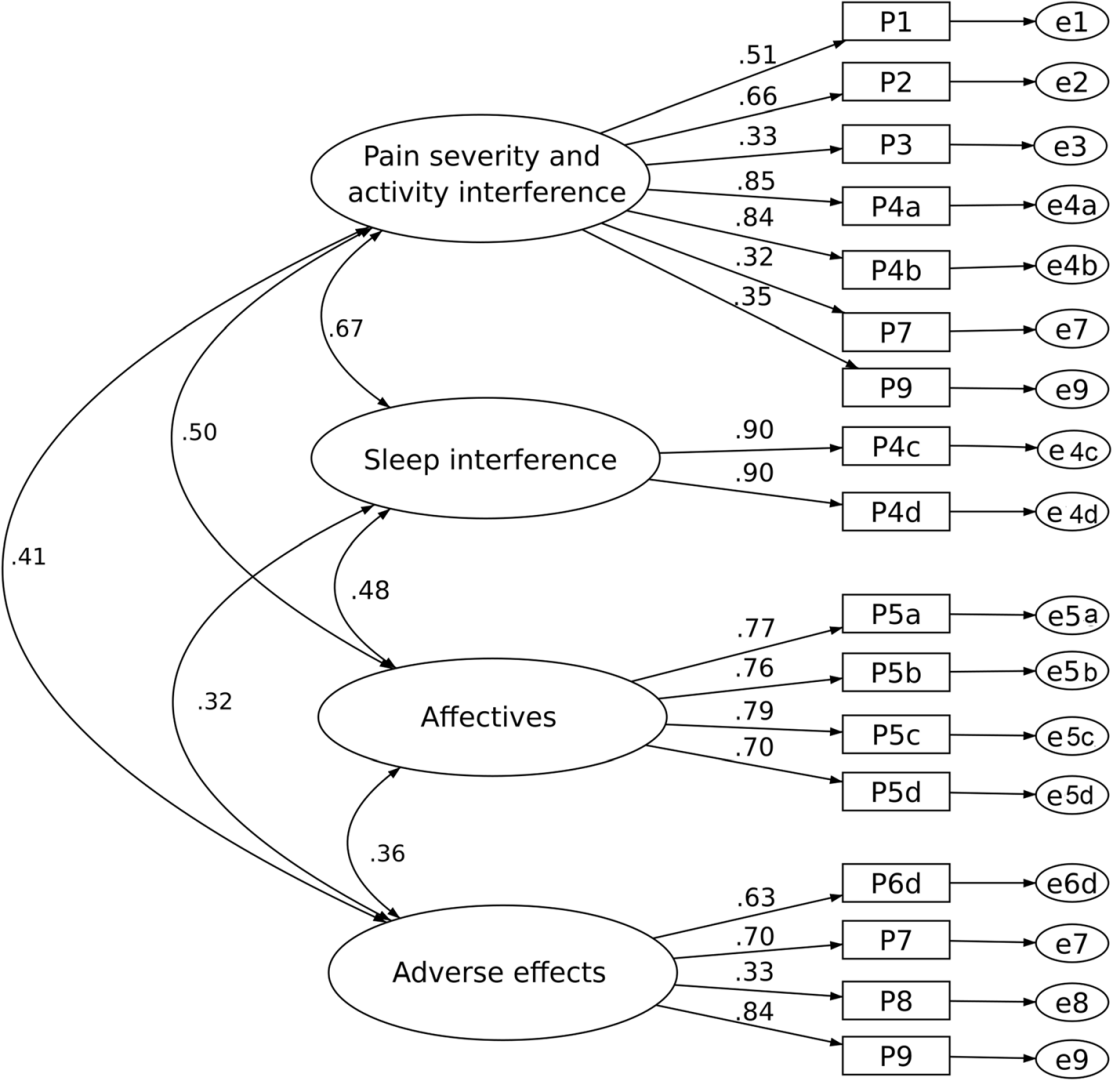
Four-factor model (Model III)

**Fig. 5:**
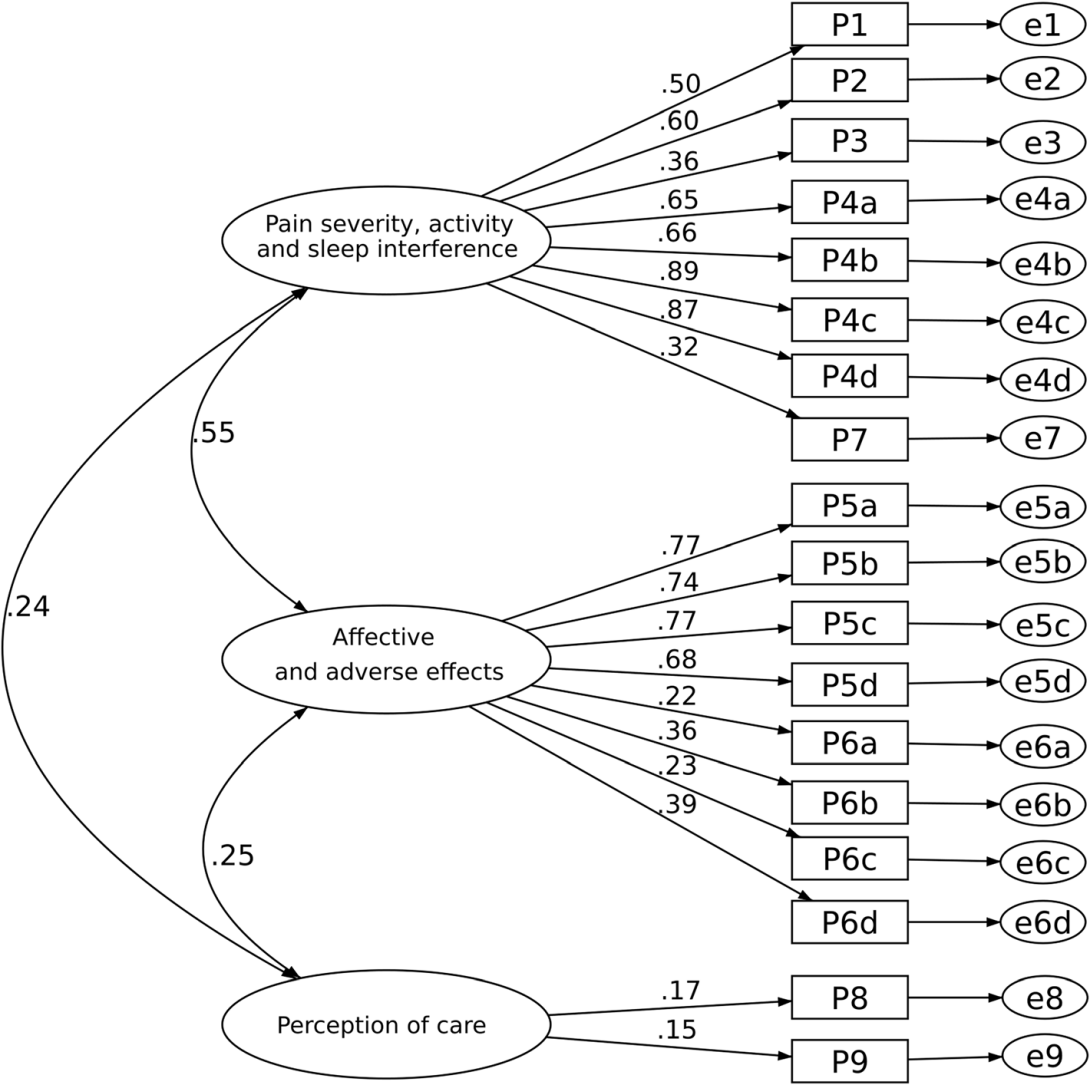
Three-factor model (Model IV)

Model III (Fig. 4) was based on the four-factor solution from the study by Botti et al. [6] also demonstrates an acceptable model fit with small χ^2^/df (2.40), CFI of 0.922, TFI of 0.895, and RMSEA of 0.068. The fit indices for the three-factor model (Model IV), however, were not acceptable based on this dataset (Fig. 5).

Factor loadings for each of the latent factors for Model II are shown in Table 3. Three items had a factor loading of less than 0.40. These items are “Estimate of time in severe pain” and “Itchiness” for both models, as well as “Participation in decision making of pain treatment” in Model II.

**Table 3:** Standardized factor loadings for Model II (*N* = 302)

Items	Model II				
	Pain severity and sleep interference	Activity interference	Affective	Adverse effects	Perception of care
P1 Least pain in first 24 h	0.46				
P2 Worst pain in first 24 h	0.54				
P3 Estimate of time in severe pain	0.35				
P4a Pain interfered activities in bed		0.87			
P4b Pain interfered activities out of bed		0.90			
P4c Pain interfered falling asleep	0.93				
P4d Pain interfered staying asleep	0.90				
P5a Anxiety caused by pain			0.77		
P5b Depression caused by pain			0.76		
P5c Fright caused by pain			0.79		
P5d Helplessness caused by pain			0.70		
P6a Nausea				0.63	
P6b Drowsiness				0.70	
P6c Itching				0.33	
P6d Dizziness				0.84	
P7 Percentage of pain relief received					0.43
P8 Participation in decision making of pain treatment					0.21
P9 Satisfaction with pain treatment					0.67

### Descriptive data of APS-POQ-R

The items that measure the responses with a numerical rating scale from 0 to 10 were considered as continuous variables. The number of respondents, minimum, maximum, mean, and standard deviations (SD) of the pain scores are shown in Table 4.

**Table 4:** Descriptive statistics for continuous items in the APS-POQ-R

**Items**	**Scores**			
	**Minimum**	**Maximum**	**Mean**	**Standard deviation**
P1 Least pain in 24 h	0	8	2.27	1.78
P2 Worst pain in 24 h	0	10	5.34	3.52
P3 Estimate of time in severe pain (%)	0	100	19.84	23.34
P4a Pain interfered activities in bed	0	10	3.25	2.75
P4b Pain interfered activities out of bed	0	10	3.42	2.95
P4c Pain interfered falling asleep	0	10	2.07	2.68
P4d Pain interfered staying asleep	0	10	1.86	2.50
P5a Anxiety caused by pain	0	10	1.64	2.49
P5b Depression caused by pain	0	10	1.09	2.14
P5c Fright caused by pain	0	10	1.27	2.29
P5d Helplessness caused by pain	0	10	0.98	2.16
P6a Severity of nausea	0	10	1.56	2.42
P6b Severity of drowsiness	0	10	2.16	2.75
P6c Severity of itchiness	0	9	0.47	1.48
P6d Severity of dizziness	0	10	1.75	2.50
P7 Percentage of pain relief received (%)	0	100	74.44	19.84
P8 Participation in decision making of pain treatment	0	10	2.85	2.96
P9 Satisfaction with pain treatment	4	10	8.05	1.33

## Discussion

The present study examines the construct validity of the APS-POQ-R using confirmatory factor analysis, in contrast to the use of exploratory factor analysis in previous studies. Our findings support the five-factors structure that was hypothesized by Gordon et al. [5] in a multi-ethnic patient sample using the CFA approach.

The psychometric evaluation of APS-POQ-R has been performed in adult inpatients across different cultures using an exploratory factor analysis approach [7, 8, 21]. The construct validity for subscales “pain severity”, “adverse effects”, and “interference of activity and sleep” was consistent across different cohorts. The items “percentage of pain relief received”, “participation in decision making” and “satisfaction”, however, have inconsistent loadings on the latent factors across studies [6]. These three items were proposed to measure the “quality of pain management” domain from the original study by Gordon et al. [5]. The poor psychometric properties of the “quality of pain management” domain suggest that the validity and reliability of APS-POQ-R need to be evaluated before being implemented in a local clinical setting [22].

Among the hypothetical models tested in this study, two models (model II and model III) demonstrate acceptable fit indices and are superior to the one-factor model. Although Model III was based on the study by Botti et al. [6] has an overall satisfactory model fit, the latent factor of “Perception of care” is lacking. Moreover, the item “ability to participate in pain treatment” was not included in model III due to its low communality in the original study. The authors, however, suggested to retain the item and considered it as independent of APS-POR-R constructs due to its conceptual importance [6].

In recent years, patient-centered care has been a focus in the healthcare system, and patients’ needs are considered in the decision-making process [23]. Patients’ perception of care, therefore, becomes an integral component in the development of questionnaires for quality improvement purposes. As such, Model II that includes all items that are proposed to measure patients’ satisfaction and perception would be a better model than other models for the APS-POQ-R construct.

For Model II, the item “participation in decision making of pain treatment” has a standardized factor loading of less than 0.3 in our cohort. The interpretation of “participation in pain management” may be different among individuals. McTier et al. reported that most patients tended to report their pain severity rather than participating in decision-making on the treatment options [24]. Some patients may perceive that reporting pain is a form of participation, but others may prefer choosing a treatment option for themselves. To improve the representativeness of this item for the underlying latent factor, modification of the item may be considered.

The “affective” and “adverse effects” subscales are stable across previous studies [21]. The standardized factor loading for “itchiness”, however, was lower than that of other adverse effects. A few reasons may explain this finding. First, there was a high proportion of patients who did not experience this adverse effect [5]. Second, different analgesics administered to the patients may affect the manifestation of the side effects. For example, dizziness, nausea, and vomiting, as well as itchiness, are more common in opioids users than those who received paracetamol or non-steroidal anti-inflammatory drugs. The difference in the combination of analgesics administered to the patients may contribute to the lower factor loading for “itchiness”.

The satisfaction score among our participants was high, with a mean value of 8.05. The influence on the level of satisfaction of postoperative pain management could be multifactorial. The level of patient satisfaction does not only depend on the effectiveness of pain relief but is also determined by the care from the healthcare providers. An opportunity to participate in pain treatment and to gain better knowledge about the pain care that they are receiving may contribute to higher patient satisfaction [21, 25].

Our findings support the APS-POQ-R as a QI tool for pain management evaluation in this setting. This validated questionnaire can be used as a standard tool to provide a benchmark for the comparison of pain management quality within the measured patient cohort. Besides, the different subscales of the questionnaire allow clinicians or researchers to identify areas for improvement more effectively. Of note, we plan to utilize this questionnaire for our postoperative patients to enable continual evaluation and reassessment of the construct stability over time. The use of patient-reported pain experience measures will also encourage patients’ engagement in their pain care. In the future, the psychometric testing of APS-POQ-R using the CFA approach should be performed in other clinical settings such as medical patients who receive pain care to establish a standardized QI instrument for comparison of the quality of pain care.

Our study has a few limitations. First, the patients may suffer from the lingering effects, such as dizziness and sleepiness of anesthesia in the postoperative period. Hence, the recall of postoperative pain experience may be affected. Second, we recruited only gynecological patients who represent one of the main surgical subspecialties in our center. Recruitment of patients from different subspecialties in the future would enable the generalization of the study results to other surgical patients.

## Conclusions

The psychometric testing of a measurement instrument is important to support its use in research or clinical practice. The present study has demonstrated that a five-factor structure of the APS-POQ-R is the best fitting model in our multi-ethnic patient sample. This study results provide further evidence to support the use of APS-POQ-R as a measurement tool for pain management evaluation.

## Data Availability

The datasets generated and analyzed during the current study are available from the corresponding author on reasonable request.

## Abbreviations

APS-POQ-R: Revised American Pain Society Patient Outcome Questionnaire
QI: Quality improvement
CFA: Confirmatory factor analysis
EFA: Exploratory factor analysis
UMMC: University of Malaya Medical Centre
IMU: International Medical University
APS: Acute pain service
NSAIDs: Non-steroidal anti-inflammatory drugs
RMSEA: Root-mean-squared error of approximation
CFI: Comparative fit index
TLI: Tucker-Lewis Index
SD: Standard deviation
